# Lacerated Wound of the Neck

**Published:** 1881-04

**Authors:** 


II.—LACERATED WOUND OF THE NECK.—*Previous History.*—The horse was blind. The animal became frightened by the whistling of one of the engines of the elevated road; the driver lost control of the animal, and she dashed into a plate-glass window of a store on the corner of Thirty-seventh street and Second avenue; becoming more frightened, she turned completely around, and dashed into a window on the opposite side of the street before she could be controlled. On admission to the hospital, the animal was suffering from a lacerated wound in the neck midway between the head and shoulders, and extending from above downwards. The wound was thirteen inches long, and was bleeding very freely. The wound contained many fragments of glass. Several small arteries were ligated, all the particles of glass removed, the wound dressed, and the capillary oozing arrested by cold and mild astringents. The following morning the wound was carefully examined and found even more extensive under the skin than upon the surface. There was some laceration of the muscles above and anterior to the external wound. The wound was four inches deep, having penetrated some of the muscles; there was also extensive laceration of the soft parts under the skin for six or seven inches posterior to and below the open wound. A counter opening was made through the most dependent part of this pocket, and a perforated drainage-tube introduced through this counter opening and brought out at the last point of the wound, thus establishing a complete and free drainage. The original wound closed by interrupted sutures. The wound was then thoroughly washed out with a solution of carbolic acid (1 to 40). A side line was then attached to the bit and ring of the surcingle to the same side as the wound, to prevent the horse throwing the head to the opposite side, and thus increasing the tension on the sutures. The wound was thoroughly washed out twice a day with carbolic acid solution. Very little swelling followed, and the wound healed very rapidly. The drainage-tube was removed on the tenth day, and the animal returned to the owner with positive instructions to keep the side line on a few days longer. Failing to do so, one edge of the wound was torn open, but healed in a few days by granulating from the bottom. The final result was all that could be desired.

